# Commissioning and clinical implementation of low dose dual‐field rotational TSET

**DOI:** 10.1002/acm2.70180

**Published:** 2025-07-15

**Authors:** Broderick Ivan McCallum‐Hee, Mounir Ibrahim, Godfrey Mukwada, Pejman Rowshanfarzad, Joshua Dass, Jude Dewitt, Robert Parin, Gregory Withey, Zaid Alkhatib

**Affiliations:** ^1^ Department of Radiation Oncology Sir Charles Gairdner Hospital Nedlands Western Australia Australia; ^2^ School of Physics, Mathematics and Computing The University of Western Australia Crawley Western Australia Australia; ^3^ Centre for Advanced Technologies in Cancer Research (CATCR) Perth Western Australia Australia; ^4^ Department of Medical Technology & Physics Sir Charles Gairdner Hospital Nedlands Western Australia Australia

**Keywords:** dual field rotational TSET, radiation oncology, radiotherapy, total skin electron irradiation, total skin electron therapy, TSEI, TSET

## Abstract

**Background:**

Total skin electron therapy (TSET) is a specialized technique for treating cutaneous T‐cell lymphomas, such as mycosis fungoides. While traditional TSET regimens prescribe 30–36 Gy, low dose TSET at 12 Gy has gained attention due to its reduced toxicity. The dual‐field rotational TSET technique, utilizing a standard linac, offers a practical solution for improved dose distribution. However, limited studies exist on its commissioning and clinical implementation.

**Purpose:**

This study aimed to commission and clinically implement a low dose dual‐field rotational TSET technique. The primary objectives were to optimize beam geometry, characterize dosimetry, and ensure treatment uniformity while maintaining compliance with ACPSEM, EORTC, and AAPM guidance.

**Methods:**

A Varian TrueBeam linac equipped with a 6 MeV high‐dose‐rate total skin electron beam was commissioned for TSET. Dosimetric measurements included beam output calibration, relative dosimetry, and dose uniformity assessment using an ion chamber and Gafchromic film, with verification using an anthropomorphic phantom. A treatment dose calculation methodology was developed. Shielding, ozone generation, and quality assurance were assessed. In vivo dosimetry was performed for treatment validation.

**Results:**

The optimal dual‐field beam geometry was determined to be ± 18° from the horizontal, achieving vertical uniformity within ± 2.9% and an overall treatment plane uniformity of ± 8.6%. Under rotation, the depth dose is delivered 100% at the surface, with bremsstrahlung contamination of 0.2 Gy. Custom eye shielding was developed, and ozone concentrations remained below the NCRP 0.1 parts per million (ppm) safety thresholds. In vivo dosimetry confirmed treatment uniformity within EORTC guidelines and identified three regions requiring dose boosts: the shoulders, palms, and inner thighs.

**Conclusions:**

The commissioned dual‐field rotational TSET technique provides a viable treatment option, achieving clinically acceptable dose distribution.

## 1 INTRODUCTION

Total skin electron therapy (TSET) is a specialized radiation therapy technique primarily used for treating cutaneous T‐cell lymphomas, such as mycosis fungoides.^1^, ^2^ It delivers a uniform dose of low‐energy electrons to the entire skin surface, sparing deeper tissues and organs from significant radiation exposure. Traditional TSET prescription regimes recommend a total dose of 30–36 Gy; however, recent literature shows increasing interest in low dose TSET with a total dose of 12 Gy due to its advantages in reducing dose‐dependent toxicity while maintaining efficacy.^2^

Various techniques have been developed to deliver TSET, each presenting unique technical and logistical challenges^3^, ^4^ In facilities where space constraints limit the feasibility of alternative techniques, dual‐field rotational TSET using a standard linear accelerator (linac) offers a practical and effective solution. This approach employs patient rotation combined with dual‐angled fields to achieve dose coverage comparable to other techniques in significantly shorter treatment times.^3^, ^4^, ^5^, ^6^ During treatment, patients are supported by a dedicated frame and stand on a rotating platform positioned at an extended distance from the linac; the frame includes features such as a handlebar to aid stability and reproducibility.

Guidelines for TSET are available from several professional bodies, including ACPSEM (2015), EORTC (2002), and AAPM (1987).^7^, ^8^, ^9^ At the time these recommendations were published, low dose TSET was not a clinical standard, and therefore, specific guidance in this context is lacking. In summary, field uniformity should be maintained within ± 10%, the dose at 4 and 20 mm depths should be at least 80% and less than 20% of the maximum dose, respectively, and photon contamination should remain below 0.7 Gy.^7^, ^8^, ^9^

While the dosimetry and commissioning of both rotational and dual‐field TSET techniques have been previously reported, limited studies exist on the combined rotation dual‐field TSET technique.^5^, ^10^, ^11^, ^12^, ^13^, ^14^, ^15^, ^16^, ^17^ Previous works differ in multiple aspects, including treatment machine, beam energy, dose rate, treatment distance, and dose calculation methodologies.^18^, ^19^, ^20^, ^21^

This work aims to contribute to the existing body of knowledge by providing comprehensive details on the commissioning and implementation of a low dose TSET technique, for which the institutional standard prescription was 12 Gy delivered in eight fractions.

## 2 METHODS

**FIGURE 1:**
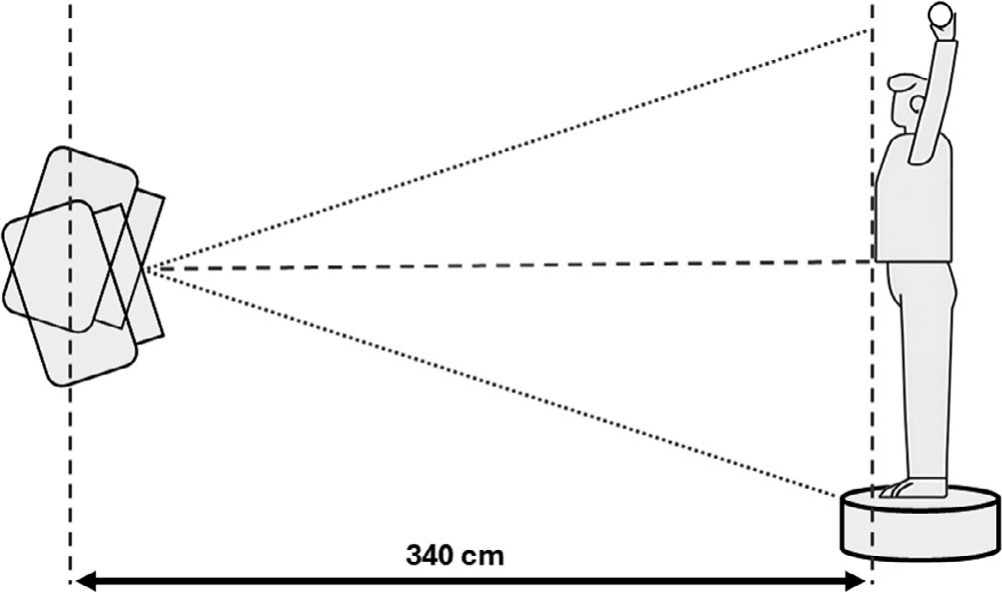
Schematic geometry of the rotational dual‐field TSET technique.

A Varian TrueBeam linac (Varian Medical Systems Inc., USA) equipped with a 6 MeV High Dose Rate Total Skin Electron (HDTSE) beam was used for all measurements. The beam was collimated to 36 cm × 36 cm by the jaws and operated at a nominal dose rate of 2500 Monitor Units (MU)/min or 25 Gy/min at a 100 cm Source‐Surface Distance (SSD).^22^ Figure 1 presents a schematic of the rotational dual‐field TSET technique.

### 2.1 OZONE AND RADIATION SHIELDING

The linac bunkers were sufficiently shielded for the clinical use of 12 MeV electron beams and 10 MV photon beams. No additional radiation shielding considerations were required given the small change in workload and use factors.^7^ Ozone is a potentially hazardous gas that can be a concern in TSET due to the use of electron beams at high beam currents over longer than standard treatment distances.^7^, ^23^, ^24^ NCRP Report 151 recommends that ozone concentrations should not exceed 0.1 parts per million (ppm).^24^ To assess this, potential ozone concentration levels were calculated using the formula from McGinley et al.^23^:

$$C=\frac{C_{o}GES_{col}Id}{NV\left(Q/V\right)}\;\times \left(1-e^{-\left(\frac{Q}{V}\right)t}\right)\times 10^{9}$$

where C is the ozone concentration in ppm, $C_{o}$ is the fraction by weight of O_2_ in air (0.232), G is the ozone production by electron irradiation of air (6 molecules per 100 eV), E is the number of electronic charges per milliamp per second of electron beam current ($6.28\times 10^{15}\;mA^{-1}s^{-1}$), $S_{col}$ is the collision stopping power of the electron (2.7 keV/cm), I is the average external electron beam current (mA), d is the electron travel distance in air (cm), N is Avogadro's number ($6.02\times 10^{23}$ molecules per 22.4 liters of air), V is the room volume in liters, Q is the room ventilation rate (liters/s), and t the irradiation time (s).

The 6 MeV HDTSE beam is operated at a dose rate of 2500 MU/min, which is 2.5 times greater than the maximum dose rate for a standard 6 MeV electron beam.^22^ The average external electron beam current was estimated to be 0.005 mA, derived by multiplying the 0.002 mA value used by McGinley for a 6 MeV electron beam by 2.5.^23^ The linac bunkers feature an open maze design without a door; however, as a conservative estimate, the room volume calculation excluded the maze. Based on construction designs, the room volume is 257 400 liters. Room ventilation was conservatively estimated to rely solely on the installed air control system, ignoring the effects of other sources of air exchange (e.g., pressure differentials). This system operates at a rate of 460 liters per second (6.4 room changes per hour). Irradiation times of both 2 h and 10 min were used as conservative considerations for commissioning measurements and treatments, respectively.

### 2.2 BEAM LINEARITY AND OUTPUT

An FC65‐G Farmer‐type ionization chamber (IBA Dosimetry GmbH, Germany) was irradiated with 100, 200, 500, 700, 1000, 1500, 2000, 5000, and 8000 MU to validate beam linearity. Linearity was evaluated based on the charge reading per MU, expressed as a percentage relative to that at 100 MU.

Beam output was calibrated at 100 cm SSD, and absolute dosimetry was determined following the IAEA TRS‐398 formalism.^25^ The beam quality index, R_50_, was determined by measuring a percentage depth ionization (PDI) curve using a plane‐parallel Roos ionization chamber type 34001 (PTW Freiburg GmbH, Germany) in the Blue Phantom 2 3D water tank (IBA Dosimetry GmbH, Germany). The PDI was converted to a percentage depth dose (PDD) by correcting for water‐air stopping power ratio variations using the multiparameter equation provided by Burns et al.^25^, ^26^, ^27^ Absolute dose was measured at the reference depth, $z_{ref}$, for a field size of 36 cm × 36 cm at the phantom surface. $z_{ref}$ in g/cm^2^ is given by the following equation^25^:

$$\;z_{ref}=0.6\;R_{50}-0.1$$

As discussed in IAEA TRS‐381 and TRS‐398, the precise choice of field size is not critical, provided it is larger than 10 cm × 10 cm, thereby maintaining broad beam conditions where the depth dose is relatively independent of field size and ensuring the applicability of stopping‐power ratio formalism.^25^, ^28^ The 6e HDTSE beam will be used exclusively at a field size of 36 cm × 36 cm, making this the most convenient reference field choice. Measurements were performed using a Roos chamber with a calibration coefficient traceable to the ARPANSA Primary Standards Dosimetry Laboratory. Beam output calibrated to deliver 1 Gy per 100 MU at a depth of dose maximum, d_max_.

### 2.3 OPTIMAL BEAM GEOMETRY

The optimal dual‐field beam geometry is the one that achieves the greatest combined field uniformity. Vertical uniformity was measured by placing a Farmer‐type ionization chamber every 5 cm along the horizontal in the central ± 30 cm and every 10 cm between ± 30 and ± 80 cm. The chamber was mounted to a large wooden board with its axis perpendicular to the vertical plane, and the manufacturer‐supplied build‐up cap was attached. Each angle from 15° to 20° was investigated. The irradiated cable length was minimized and kept consistent across all measurements to reduce cable effects.

### 2.4 DUAL‐FIELD UNIFORMITY

The uniformity of the dual‐field beam geometry was characterized across the central 180 cm × 60 cm area using Gafchromic EBT3 film (Ashland, USA). EBT3 film has been previously validated for TSET dosimetry.^29^ A total of 133 pieces of approximately 2 cm × 3 cm film were placed equidistantly across the area with a horizontal and vertical spacing of 10 cm (19 rows and 7 columns). Films were scanned the day after irradiation at 300 dpi resolution in 48‐bit RGB mode using an Expression 1200XL scanner (Seiko Epson Corporation, Japan) and analyzed with SNC Patient software (Sun Nuclear Corporation, USA). To minimize lateral response artifacts and scanner non‐uniformities, films were scanned simultaneously in the same orientation along the central axis of the scanner bed in batches of up to 19 (column by column for uniformity measurements). The applied film calibration was created by placing film pieces at d_max_ for 100 cm SSD and irradiating them with doses from 4 to 400 cGy. SNC Patient uses single‐channel dosimetry (in this study, the red channel was selected) and applies an exponential fit to the calibration data. Readings were taken as the average of the central 1 cm × 1 cm region of each film, excluding edges and areas where tape had been attached.

### 2.5 TSET CALIBRATION POINT DOSE

The TSET calibration point was set at the location of d_max_ for the dual‐field geometry at an SSD of 340 cm. Measurements were performed using a Roos chamber and an RW3 solid water slab phantom (PTW Freiburg GmbH, Germany), following a methodology analogous to that employed by Schiapparelli et al.^12^ The mass density of the solid water was measured using a calibrated digital caliper, metal ruler, and scale. As outlined in the TRS‐398 formalism, measurements made in plastic phantoms can be related to those in water via scaling depths and fluences.^25^ To determine the depth scaling factor, $c_{pl}$, the below relationship was used^30^:

$$\;c_{pl}=\frac{R_{50,ion,w}}{R_{50,ion,pl}}$$

where $R_{50,ion,w}$ is the half value of the PDI in water and $R_{50,ion,pl}$ is the half‐value of the PDI in plastic, both expressed in terms of density thickness. The PDI in water at 100 cm SSD was measured as described in the “Beam Linearity and Output” section. Under the same setup geometry, the PDI in plastic was measured at a 1 mm resolution. The fluence‐scaling factor, $h_{pl}$, was subsequently determined via the below relationship^30^:

$$\;h_{pl}=\frac{M_{Q,w}\left(z_{w}\right)}{M_{Q,pl}\left(z_{pl}\right)}$$

where $M_{Q,w}\left(z_{w}\right)$ and $M_{Q,pl}\left(z_{pl}\right)$ are the influence quantity corrected measurements for those obtained in water at $z_{w}$ (specifically $z_{ref}$) and at the equivalent depth in plastic, $z_{pl}$. The depth $z_{pl}$ is related to $z_{w}$ as follows^25^:

$$\;z_{pl}=\frac{z_{w}}{c_{pl}}$$

The PDI in solid water at 340 cm SSD for a single horizontal beam produced by a gantry angle of 270° (perpendicularly incident) and the dual‐field geometry was measured in 1 mm steps. The dual‐field representative PDI was obtained by summing contributions from each beam. PDDs were obtained by scaling depths by $c_{pl}$, applying $h_{pl}$, and correcting for variations in the water‐to‐air stopping power ratio. Between the surface and $z_{ref}$, a linear fit from a value of unity to the measured $h_{pl}$ was used to account for depth dependence.^25^

Using the PDD‐derived value for R_50_, $c_{pl}$ and $h_{pl}$ the beam output for the single horizontal beam at $z_{ref}$ in water, $D_{h}$, was determined using the following equation:

$$\;D_{h}=\left[M_{Q,pl}\left(Z_{pl}\right)\cdot h_{pl}\right]\cdot N_{D,w,Q_{cross}}\cdot k_{Q,Q_{cross}}$$

where $N_{D,w,Q_{cross}}$ is the calibration coefficient and $k_{Q,Q_{cross}}$ the beam quality correction factor. The dose at the TSET calibration point, $D_{TSET}$, was then related to $D_{h}$ through a ratio factor, A ^12^:

$$\;D_{TSET}=A\cdot D_{h}$$

where A is the ratio of the measured ionizations collected from irradiation of the two geometries provided the beam qualities are comparable. The resulting $D_{TSET}$ and A factor were validated using film irradiated under identical conditions. Absolute film dosimetry was conducted using the method described in the previous section.

### 2.6 DOSE RATE SSD DEPENDENCE

Although the rotational technique aims to minimize SSD variations, they may occur due to patient geometry, motion, and treatment setup. Dose rates for the dual‐field beam from 310 to 370 cm SSD, relative to 340 cm SSD, were measured in solid water using a Roos chamber at $z_{ref}$.

### 2.7 PRESCRIBED DOSE

The prescribed dose, defined at the surface of a cylinder, was related to the TSET calibration point via a multiplication factor, B. The factor B was equal to the dose at the surface divided by that at the calibration point. Measurements were performed using film and an available 3D‐printed, tissue‐equivalent cylinder phantom consisting with a diameter 27 cm and a height 20 cm.^31^ The phantom was setup to 340 cm SSD and rotated through the dual‐field geometry, ensuring each beam's duration matched to a single rotation. Surface measurements were taken as the average from four locations spaced 90° apart around the circumference to minimize the impact of setup uncertainties.

### 2.8 END TO END MEASUREMENTS AND EYE SHIELDING

**FIGURE 2:**
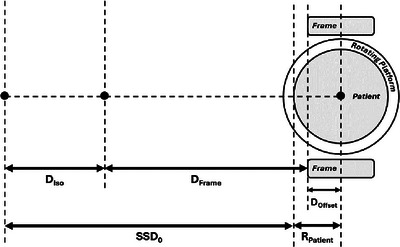
Schematic of distances for the frame distance calculation.

The required MU for each beam per fraction, $MU_{beam}$, was determined as follows:

$$MU_{\mathrm{beam}}=\frac{D_{\mathrm{Frac}\mathrm{tion}}}{B\cdot D_{\mathrm{TSET}}}$$

where $D_{Fraction}$ is the dose per fraction. The beam‐on time was determined by dividing by the dose rate. To deliver the specified dose evenly across the patient surface, rotation had to be a multiple of the calculated beam‐on time. To maximize patient comfort, the time for a single full rotation was matched to the beam‐on time. For patient setup, their average circumference, $C_{Patient}$, and radius, $R_{Patient}$, were calculated by taking measurements at the shoulders, chest, waist and hips. The setup procedure involved positioning the frame using a laser distance meter QM‐1626 (Digitech, China) at an isocenter to frame edge distance, $D_{Frame}$, calculated as follows:

$$\;D_{Frame}=SSD_{0}-D_{Iso}+\frac{C_{Patient}}{2\pi }-D_{Offset}$$

where $SSD_{0}$ is the nominal SSD of 340 cm, $D_{Iso}$ is the source‐to‐isocenter distance of 100 cm and $D_{Offset}$ is the frame‐to‐platform rotational axis offset. A schematic of the distances is presented in Figure 2.

For eye shielding, a modified version of the commercial Centrist lead glasses (ProTech Medical, USA) with double lenses, equivalent to 1.5 mm Pb, was used. PDD and eye shielding measurements were obtained using the Alderson RANDO Phantom (Radiology Support Devices Inc., USA) with 12 pieces of film in the central abdomen region and two under the eyes. The film for the PDDs was cut using a laser cutter to match the phantom's geometry and sandwiched between the slices of the RANDO phantom. Bremsstrahlung contamination was evaluated based on the PDD value at a depth of 25 mm.^21^

Surface dose assessment was conducted using a 3D‐printed phantom, with results described in the related study by Lee et al.^31^

### 2.9 QUALITY ASSURANCE

A quality assurance (QA) program incorporating patient‐specific and routine checks was implemented to ensure consistency with commissioning. A qualified medical physicist reviewed all dose and frame positioning calculations, as these are both crucial and not conducted in a treatment planning system. Patient rotational speed, which is also critical to ensure full and even surface coverage, was verified in a dry run prior to each fraction. Daily beam output and uniformity constancy were monitored using the Varian Machine Performance Check (MPC). As measurements in TSET conditions are difficult and time‐consuming, baseline values for the beam at 100 cm SSD were established. Monthly output constancy and annual output measurements were performed in solid water and water, respectively. Monthly energy constancy was assessed using a ratio of ionization measurements, as suggested by IPEM 81, using solid water build‐up amounts of 12 and 25 mm.^32^ Monthly profile constancy was performed using the 2D IC Profiler array (Sun Nuclear Corporation, USA). Annual PDD and profile checks were conducted in a water tank. Machine mechanical checks, such as gantry and collimator angle accuracy, are not TSET‐specific and were already a part of routine QA.

### 2.10 PATIENT IN VIVO DOSIMETRY

Given the specialist nature of the technique, in vivo, dosimetry was performed for the first fraction of each patient. The film was placed at 24 locations across the patient.

## 3 RESULTS

### 3.1 Ozone and radiation shielding

The ozone concentration after 10 min was calculated to be 0.021 ppm, and after 2 h, 0.032 ppm. After an infinitely long irradiation, the ozone concentration would not exceed 0.032 ppm.

### 3.2 Beam linearity and output

The beam was linear within 0.14% between 100 and 8000 MU.

The 6e HDTSE beam R_50_ at 100 cm SSD was 2.35 g/cm^2^ and corresponding to a z_ref_ of 1.31 g/cm^2^. The PDD correction from z_ref_ to d_max_ at 1.28 g/cm^2^ was 1.006. Measurements were corrected for temperature and pressure, the polarity effect (k_pol_ = 1.001) and ion recombination (k_s_ = 1.009). Beam output was monitored during all commissioning measurements, and an upwards drift of approximately 1% over 3 months was observed, which is a similar trend to other treatment beams.

### 3.3 Optimal beam geometry

**FIGURE 3:**
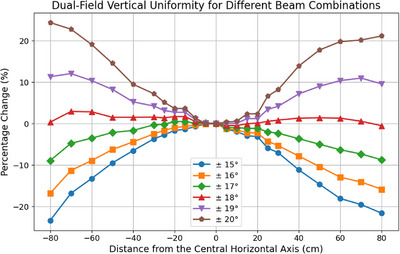
Dual‐field vertical uniformity for all measured beam combinations as a percentage change relative to the central horizontal axis.

The dual‐field vertical uniformity for all symmetric beam combinations, expressed as a percentage change relative to the central horizontal axis, is presented in Figure 3. While asymmetric beam combinations were also checked, they did not produce improved uniformities. The combination of beams ± 18° from the horizontal produced the highest vertical uniformity, with a maximum measured deviation of 2.9%.

### 3.4 Dual‐field uniformity

**FIGURE 4:**
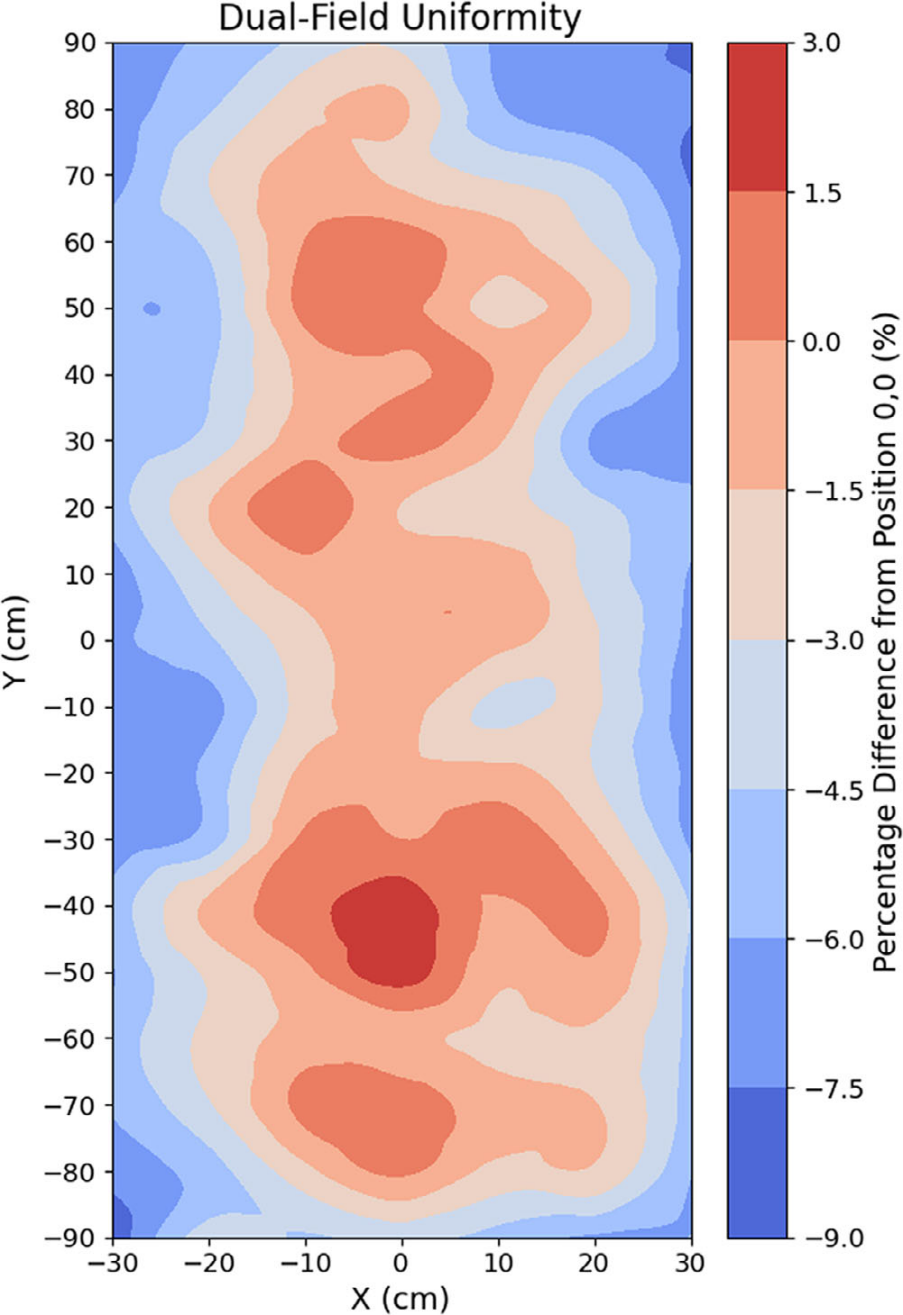
Film measured dual‐field uniformity as a percentage change relative to the center from 133 pieces of approximately 2 cm × 3 cm film placed equidistantly across the area.

Results from the dual‐field uniformity are presented in Figure 4. Uniformity within ± 4% was observed along the central vertical axis. Across the entire measured region, the maximum deviation observed was −8.6% at the upper right corner of the field. Along the vertical axis, between −80 and +80 cm, the maximum deviation was 2.8%.

### 3.5 TSET calibration point dose

The RW3 solid water had a mass density of 1.045 g/cm^3^, a c_pl_ of 0.969 and an h_pl_ of 1.017. The single horizontal beam at 340 cm SSD had an R_50_ of 2.11 g/cm^2^ and z_ref_ of 1.17 g/cm^2^, equating to an equivalent z_ref,pl_ of 1.20 g/cm^2^ (11.5 mm). Given the available solid water and water‐equivalent depth of the Roos chamber, measurements were taken at a depth of 11.3 mm.^25^ Measurements were corrected for temperature and pressure, the polarity effect (k_pol_ = 1.002), and ion recombination (k_s_ = 1.002). The dose rate, determined via ionization measurements; was D_h_ 0.069 cGy/MU. Absolute film measurements agreed within 1.4%.

**FIGURE 5:**
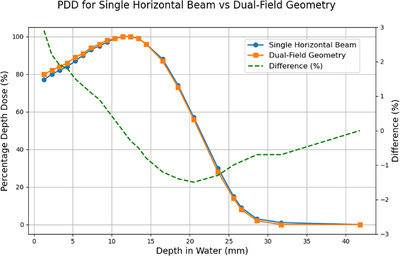
PDD for the single horizontal beam and the dual‐field geometry.

PDDs for the single horizontal beam and dual‐field geometry are presented in Figure 5. The ratio factor A was 0.957 ± 0.002, and D_TSET_ was 0.06566 cGy/MU. Validation of the A factor via film agreed within 0.21%.

### 3.6 Dose rate SSD dependence

When the measured dose rate dependence data was fitted to a power function, it resulted in a fit with a coefficient of determination of 1.0, of the form:

$$D=D_{0}\;{\left(\frac{SSD_{0}}{SSD}\right)}^{2.9}$$

where *D* is the dose at the *SSD* of interest, and *D*_0_ the dose at a nominal *SSD*_0_ of 340 cm.

### 3.7 Prescribed dose

The factor B, defined as the dose at the surface divided by that at the calibration point was 0.434 ± 0.008. Dose calculation validation measurements with film, using this factor, agreed within 2%.

### 3.8 End to end measurements and eye shielding

**FIGURE 6:**
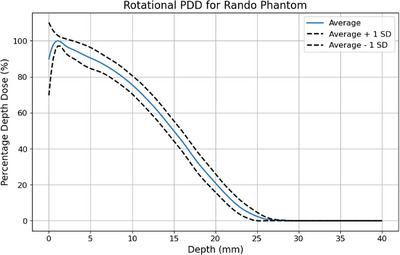
Rotational PDD for the RANDO phantom with average and ± 1 standard deviation (SD) from the twelve measurements.

The measured PDD for the RANDO phantom is presented in Figure 6. The eye shielding transmission was 17 ± 1% or 2.0 ± 0.1 Gy for a full treatment course. The bremsstrahlung contamination was 2 ± 2% or 0.2 ± 0.2 Gy for a full treatment course at a depth of 25 mm.

### 3.9 Quality assurance

Patient dose and frame positioning calculations were performed in a read‐only Excel sheet (Microsoft, USA), saved as a PDF, and uploaded to the department's record and verify system. Document review by a physicist was similarly recorded in the system. For the standard departmental prescription of 1.5 Gy per fraction, 5264 MUs per field were delivered over 126.3 s. The platform speed per single rotation was set to the same value, with a ± 1.5 tolerance and ± 2 action level. Varian MPC baselines were established after beam output adjustment in water. The energy constancy ionization ratio baseline was 0.28. The monthly QA baseline profile was measured for a 36 cm × 36 cm field at 70 cm SSD with 13 mm of build‐up material (including the amount added plus device inherent material). This SSD was selected to sufficiently capture the beam penumbra. Annual PDD and profile baselines were measured using an electron diode.

### 3.10 Patient in vivo dosimetry

Patient in vivo dosimetry results from 10 measurement sessions across six different patients are presented in Table 1. The top of the head, elbows, and top of feet received doses above 110%, while the shoulders, palms, and inner thighs received doses lower than 90%.

**TABLE 1:** In vivo dosimetry results. In vivo results are given with the 1 standard deviation uncertainty.

Location	In vivo result (cGy)	Difference from 150 cGy (%)
Top of head (rear)	176 ± 27	17%
Top of head	196 ± 38	31%
Top of right shoulder	122 ± 34	−19%
Top of left shoulder	127 ± 37	−16%
Right elbow	202 ± 26	35%
Left elbow	192 ± 9	28%
Right hand (middle dorsum)	153 ± 48	2%
Right hand (upper middle palm)	105 ± 51	−30%
Left hand (middle dorsum)	145 ± 28	−3%
Left hand (upper middle palm)	106 ± 37	−30%
Right axilla	153 ± 21	2%
Left axilla	147 ± 26	−2%
Upper thorax	142 ± 12	−5%
Upper back	148 ± 9	−1%
Anterior abdomen	153 ± 13	2%
Lower back	137 ± 9	−9%
Right abdomen	152 ± 5	1%
Left abdomen	154 ± 12	3%
Right upper medial thigh	127 ± 21	−15%
Left upper medial thigh	122 ± 16	−18%
Right buttock	162 ± 7	8%
Left buttock	158 ± 9	6%
Right foot (middle dorsum)	203 ± 15	35%
Left foot (middle dorsum)	195 ± 6	30%

## 4 DISCUSSION

### 4.1 Ozone and radiation shielding

All calculated ozone concentration levels, including those for infinitely long irradiation, remain well within the NCRP tolerance of 0.1 ppm, ensuring no safety concern.^24^ After an irradiation of 10 min, some individuals might detect the presence of ozone, as it becomes noticeable at concentrations of 0.015–0.02 ppm.^33^ However, the calculations are intentionally conservative (small room volume, low ventilation rate, and long irradiation time), making this detection unlikely. Both patients and staff can be reassured of their safety. Further supporting this low risk, an experimental study by Hara et al., which measured a concentration of approximately 0.006 ppm for a room with half the air replacement rate using a similar electron beam.^34^

### 4.2 Beam linearity and output

Varian TrueBeam functional performance characteristics state a beam linearity deviation of ± 1% for electron beams ranging from 0.5 Gy (50 MU) to 100 Gy (10 000 MU).^22^ The measured linearity is well within these specifications, confirming its suitable for use.

Although the output drift for a Varian TrueBeam 6eHDTSE beam has not been previously reported, a multi‐institutional study by Glide‐Hurst et al. quantified the temporal stability for non‐HDTSE electrons and photons.^35^ The 6eHDTSE beam output upward drift is not unique and has been observed across all beam energies on the TrueBeam linac, potentially attributable to changes in linac dose monitoring capabilities.^35^, ^36^

If this 1% drift per 3 months continues consistently, it could inform QA program optimization and identify changes in machine behavior. For example, it could help reduce the required frequency of comprehensive output checks, as recommended in guidelines such as AAPM TG‐198 and MPPG 8.b.^37^, ^38^ However, further long‐term data from multiple machines, along with a comprehensive risk analysis as described in AAPM TG‐100, would be needed to draw firm conclusions.^39^

### 4.3 Optimal beam geometry

The vertical uniformity for the chosen optimal geometry of ± 18° is well within the 10% EORTC recommendation.^8^ Previous work for similar treatment setups has also found these angles to be optimal.^16^, ^20^ This uniformity is particularly important for rotational TSET, as patient rotation minimizes the impacts of horizontal inhomogeneities. For the Modified Stanford technique, which does not benefit from patient rotation, ACPSEM/AAPM recommend a 4% horizontal uniformity, a standard which the measured vertical uniformity results also meet.^7^, ^9^ Previously reported vertical uniformities for similar geometries range from 6% to 4% when using a Perspex screen in the beam path.^18^, ^19^, ^20^ Andreozzi et al. used Cherenkov imaging to explore TSET geometries, proposing ± 19° as the optimal solution to achieve the lowest coefficient of variation in the 2D patient region.^40^ The different optimal solution observed in this study may be due to the use of vertical uniformity as the key metric.

In all vertical profiles, a slight asymmetry of up to 3% was noted, with a greater dose below the horizontal axis. This can be attributed to scatter radiation from the floor, a similar observation made by Hensley et al.^20^ The unevenness observed in measured uniformities may also result from slight chamber positioning errors or non‐perpendicularity of the measurement plane.

### 4.4 Dual‐field uniformity

The overall dual‐field uniformity remained within the 10% EORTC recommendation, with maximal deviations up to 8.6% observed at the corners of the measurement plane.^8^ Although patient geometry will vary, typically, the head and legs will have the lowest width facing the beam, while the central region will be thicker.^41^ Therefore, non‐uniformities at the corners of the plane are of less concern. For example, the average Australian has a waist diameter of around 30 cm, where even greater uniformity was observed.^42^ The uniformity along the vertical axis was similar in magnitude to that found via the chamber, further supporting the good vertical uniformity of this geometry.

### 4.5 TSET calibration point dose

The single beam dose rate, ratio factor, and calibration dose rate measured using both the ionization chamber and film were within 1.4% agreement. The agreement further supports the accuracy of this method, as confirmed by Schiapparelli et al.^12^ The PDD for the single horizontal beam and dual‐field geometry agreed within 1.5% except in the surface region, particularly after reaching the d_max_ position. The slight increase of up to 3% in PDD at shallow depths may be explained by the oblique incidence of the beam, increasing the beam path lengths at these depths. At deeper depths, the scattering, summation, and attenuation of the two beams lead to a comparable PDD.

### 4.6 Dose rate SSD dependence

The SSD dependence, as demonstrated by the coefficient of determination, can be well described by a 2.9 power function. Using the derived relationship, it can be calculated that to maintain a dose difference within ± 1%, the SSD must be kept between 338.8 and 341.2 cm. This led to a frame setup tolerance of 340 ± 1 cm for patient treatment. Ming et al. empirically determined a slightly lower SSD dependence when using ± 20° beams, with a power law of 2.25 relative to a reference SSD of 332 cm.^18^ In an ideal setup, minimal SSD dependence would mitigate the impact of patient geometry; however, a certain amount of dependence is unavoidable.

### 4.7 Prescribed dose

The B factor was determined enabling the calculation of surface dose during rotation. Previous work Ming et al. and Gaspar‐Olvera et al. found rotational‐to‐calibration point factors of 0.39 and 0.4, respectively.^18^, ^19^ These values are equivalent to multiplying the A and B factors in this study, giving a combined value of 0.415 ± 0.009. While slight differences exist, these can be attributed to variations in geometry.

### 4.8 End‐to‐end measurements and eye shielding

The measured PDD and Bremsstrahlung contamination meet recommendations for TSET and displays a shift towards the surface, as opposed to stationary PDDs.^7^, ^8^, ^9^ The measured Bremsstrahlung contamination aligns with previously reported values of between 1% and 7%.^18^, ^19^, ^20^, ^21^ The results are limited by large measurement uncertainties in the dose range of film.

As a 6 MeV beam was used without any form of beam degradation, the depth characteristics are most suitable for patients with deeper treatment depths. Hensley et al. and Piotrowski et al. utilized plastic degraders with thicknesses of 6 mm and 1 cm, respectively, to degrade the beam, resulting in shallower PDDs.^20^, ^21^ While degraders reduce the beam energy, they come with additional costs, quality control requirements and increase treatment complexity. Future work at the center will explore the addition of a degrader to support the treatment of a broader range of patient indications.

The used eye shielding had beam transmission for a full treatment course within the 5.4 Gy limit recommended by EORTC.^8^ In contrast to alternative solutions such as internal eye shields or lead shielding, patient vision was not compromised, increasing comfort and minimizing fall risk while rotating.

### 4.9 Quality assurance

The QA program was successfully implemented into routine practice. For the average Australian waist circumference of 95.1 cm, the platform speed tolerance and action levels correspond to an overlap or gap of 1.1 and 1.5 cm, respectively.^42^ If these areas of over‐ or under‐dose were consistent across all fractions, this could lead to poor treatment outcomes. To mitigate this risk, patients were positioned differently for each of the 16 beams (one lower and one upper for eight fractions of 1.5 Gy).

### 4.10 Patient in vivo dosimetry

Overall, uniformities within 9% across the patient surface were observed, except for overdoses at the top of the head, elbows, and top of the feet, and underdoses at the shoulders, palms, and inner thighs. Significant fraction‐to‐fraction and patient‐to‐patient variations were also noted, as indicated by the large uncertainties. These results are consistent with findings from similar TSET geometries. Ming et al. reported that the regions requiring boost fields varied between patients.^18^ Generally, underdosed regions included the soles of the feet, palms of the hands, top of the head, and upper parts of the arms. Hensley et al. observed dose deviations at the extremities and parts of an anthropomorphic phantom with a smaller diameter, where rotation is slower, and regions are exposed to the beam maximum for longer periods.^20^ Piotrowski et al. also measured similar regions of underdose and outlined recommendations for additional local fields.^21^

The regions of underdose requiring boost fields can generally be explained by the nature of the technique. For example, the dose to the shoulder is delivered via highly oblique beams, and this region is partially shielded by the forearms. The palms and inner thighs are shielded by the patient support handlebar and the opposite leg, respectively. As the patient rotates, their arms flared slightly outwards, resulting in a shorter SSD and higher doses. The dose to the feet is increased by scatter from the floor, the patient frame, and beam angles. The higher doses measured at the top of the head, in contrast to both previous studies and phantom measurements, can be explained by the films being attached to the hair, rather than directly on the scalp.^18^, ^21^, ^31^ This placement exposed the films to the beam maximum for the entire treatment.

### 4.11 Limitations

A limitation of this study was the range of dosimetric systems available at the center. Although GafChromic EBT3 film was used, the newer EBT4 film has demonstrated improved accuracy and may have reduced dosimetric uncertainties.^43^ Additionally, film analysis was performed using single‐channel dosimetry due to software constraints, despite recent literature supporting the advantages of multi‐channel dosimetry in reducing artifacts and improving accuracy.^44^

The study would have benefited from thermoluminescent dosimeters (TLDs), which are commonly employed in TSET.^21^ However, before clinical implementation, an independent audit utilizing TLDs was conducted, and the results agreed with the film measurements. Similarly, although ozone concentration was estimated through calculations, direct measurements such as those performed by Hara et al., would have been preferred if the appropriate equipment had been available.^34^

Another limitation was the requirement for accurate positioning of phantoms, detectors, and the platform at extended SSDs across numerous vertical and horizontal positions. In future work, this could be mitigated by employing custom‐designed measurement tools fixed to the treatment room, such as a rigid board secured to the floor or ceiling at the appropriate distance.

## 5 CONCLUSION

The commissioned dual‐field rotational TSET technique utilizes a Varian TrueBeam with the 6 MeV HDTSE beam in a standard treatment bunker. For an optimal dual‐field beam geometry with the gantry angled at ± 18° from the horizontal, a vertical uniformity within ± 2.9% and an overall treatment plane uniformity of ± 8.6% were obtained. A method for calculating the dose at a TSET calibration point and surface using a variable treatment frame setup was successfully implemented and validated. Considerations included bremsstrahlung contamination, eye shielding, radiation shielding, ozone levels, and the QA program. In vivo dosimetry validated the treatment technique and identified regions requiring boost fields, including the shoulders, palms, and inner thighs. Potential hotspot regions, also identified through in vivo measurements for management, include the elbows and feet. To support the treatment of a broader patient population, methods to reduce the beam energy, such as a beam degrader, may be beneficial.

## AUTHOR CONTRIBUTIONS

All authors contributed to the study conception and design. Data collection and analysis were performed by Broderick Ivan McCallum‐Hee, Mounir Ibrahim, Pejman Rowshanfarzad, and Zaid Alkhatib. Device engineering was performed by Jude Dewitt, Robert Parin, and Gregory Withey. All authors reviewed and discussed the results. Joshua Dass and Godfrey Mukwada provided clinical insights. The first draft of the manuscript was written by Broderick Ivan McCallum‐Hee and all authors commented on previous versions of the manuscript. All authors read and approved the final manuscript.

## CONFLICT OF INTEREST STATEMENT

The authors declare no conflicts of interest.
